# From immune equilibrium to immunodynamics

**DOI:** 10.3389/fmicb.2022.1018817

**Published:** 2022-11-25

**Authors:** Xiaoping Chen

**Affiliations:** ^1^State Key Laboratory of Respiratory Disease, Center for Infection and Immunity, Guangzhou Institutes of Biomedicine and Health, Chinese Academy of Sciences, Guangzhou, China; ^2^CAS Lamvac (Guangzhou) Biomedical Technology Co., Ltd., Guangzhou, China

**Keywords:** immune equilibrium, immune power, immune force, immune braking force, immunodynamics

## Abstract

**Objective:**

The immunology field has long been short of a universally applicable theoretical model that can quantitatively describe the immune response, and the theory of immune equilibrium (balance) is usually limited to the interpretation of the philosophical significance of immune phenomena. Therefore, it is necessary to establish a new immunological theory, namely, immunodynamic theory, to reanalyze the immune response.

**Methods:**

By quantifying the immune dynamic equilibrium as the ratio of positive and negative immune power, the immune dynamic equilibrium equation was established. Then, the area under the curve of the positive and negative immune power was assumed to be equal in the whole process of immune response (regardless of correct or not), and through thought experiments based on this key hypothesis, a series of new concepts and expressions were derived, to establish a series of immunodynamic equations.

**Results:**

New concepts of immune force and immune braking force and their expression equations, namely, the theoretical equations of immunodynamics, were derived through thought experiments, and the theoretical curves of immunodynamics were obtained according to these equations. *Via* the equivalent transformation of the theoretical equations and practical calculation of functional data, and by the methods of curve comparison and fitting, some practical equations of immunodynamics were established, and these practical equations were used to solve theoretical and practical problems that are related to the immunotherapy of infectious diseases and cancers.

**Conclusion:**

The traditional theory of immune equilibrium has been mathematized and transformed from a philosophical category into a new concrete scientific theory, namely the theory of immunodynamics, which solves the dilemma that the traditional theory cannot guide individualized medical practice for a long time. This new theory may develop into one of the core theories of immunology in the future.

## Introduction

For decades, the evolution of immunology in leaps and bounds, and the success of cancer immunotherapy in recent years has revolutionized the basic research and clinical application of immunology from just preventive vaccines for infectious diseases in the past to current immune therapeutics for various diseases. Immunology has almost been active in the entire life science and medicine. The connotation of immunology is very rich, but its core principle seems to be summed up as an immune recognition (or immunorecognition) theory. The theory of immune recognition has developed greatly from the mechanism by which the immune system recognizes self and non-self (del Guercio, 1993; Joyce and Ternette, 2021), to the pattern recognition mechanism of innate immunity (Janeway and Medzhitov, 2002; Kubelkova and Macela, 2019), and then to the generation and selection mechanism of T cell and B cell receptor repertoire of adaptive immunity (Minervina et al., 2019; Werner et al., 2021). Although the theory of immune equilibrium (or immune balance) has been recognized for a long time, it has not become the core principle of immunology (Swiatczak, 2014), because it is always bound to the philosophical analysis of the function of the immune system. The embryonic form of the immune equilibrium theory was first proposed by Élie Metchnikoff more than a century ago, that is, physiological inflammation is a physiological process necessary for the body to maintain harmony (balance) (Swiatczak, 2014; Eberl, 2016), and later developed into the immune equilibrium theory in which pro-inflammatory and anti-inflammatory responses in the immune system contend with each other (Cicchese et al., 2018). Some researchers consider that immune equilibrium is an ecological balance between the immune system and environmental factors, especially the gut microbiota (Swiatczak, 2014). The functions of the immune system can be divided into innate and adaptive immunity, or antibody-mediated (humoral) and cell-mediated (cellular) immunity (Marshall et al., 2018). With the advances in immunology research, it is generally believed that the immune system can adjust its effector function and respond to the challenge of different kinds of microorganisms in the best way. Based on the new understanding of different innate lymphoid cell (ILC) lineages and effector T cells, cell-mediated immune responses are currently classified into three main types. The type 1 cellular immune response consists of ILC1s cells expressing T-bet1 and IFN-γ, CD4^+^ Th1 cells, and cytotoxic CD8^+^ Tc1 cells, which activate mononuclear phagocytes against intracellular infected pathogens (including viruses, intracellular parasitic bacteria and protozoa). Type 2 cellular immune response consists of ILC2s, CD4^+^ Th2 cells, and CD8^+^ Tc2 cells expressing GATA-3, IL-4, IL-5, and IL-13, which induce the activation of mast cells, basophils, and eosinophils to produce IgE antibodies that counter the challenges of helminthes and venoms. Type 3 cellular immune responses are mediated by ILC3s, Th17, and Tc17 cells expressing retinoic acid-associated orphan receptors (γt^+^) and IL-17 and IL-22 (one or both). These cells activate mononuclear phagocytes but also recruit neutrophils and induce an antibacterial response of epithelial cells against bacterial and fungal infections outside the cells. On the other hand, type 1 and type 3 cellular immune responses can mediate autoimmune diseases, while type 2 responses can cause allergic diseases (Annunziato et al., 2015). In recent years, the theory of immune equilibrium has been further developed. For example, Gerard Eberl proposes that immune equilibrium is a balance state achieved by the mutual inhibition of the three (or even four) different types of cellular immune responses mentioned above (Eberl, 2016). Recently, Daniil Shevyrev et al. proposed that immune equilibrium highly depends on the interaction between antigen presentation and recognition, trying to unify the immune recognition theory and immune equilibrium theory (Shevyrev et al., 2021).

Although the present theory of immune equilibrium can well explain the philosophical significance of immune function, it cannot accurately quantitatively analyze the process of immune response, so it cannot guide the individualized immunotherapy in clinic. In the process of logically combing immunology as a whole, I found that on the basis of the traditional theory of immune equilibrium, through thought experiments and concept renewal, and through rigorous logical reasoning and mathematical operation, a new immunology theory, namely the theory of immunodynamics, can be formed. The establishment of this new theory can not only promote the progress of immunology research, but also guide clinical practice. This paper proposes the decomposition of traditional immune function or immunity into interrelated and independent parts, namely, immune reserve, immune power, immune force, and immune braking force. A series of immunodynamic equations can be established by using these new concepts, so that the immunodynamic theory can be established. Then we can use these immunodynamic equations to solve theoretical and practical problems. For example, immunodynamic equations can be used to guide the individualized treatment for patients. Especially at present, in the global pandemic of COVID-19 (Leisman et al., 2020), correct immunodynamic equations are especially needed to guide the treatment of severe and critically ill patients, such as what kind of severe or critically ill patients should be treated with immunosuppressive drugs such as corticosteroids. The same problem exists in the field of cancer immunotherapy. For example, during immune checkpoint blockade therapy, some cancer patients experience a rapid progression of the disease, known as hyperprogression, which leads to rapid death without knowing why (Han et al., 2020). We need to find the cause so that we can come up with correct countermeasures. In this paper, I propose that the immunodynamic equations can solve these important medical problems.

## Methods and results

### Theoretical model of immunodynamics

According to the traditional theory of immune equilibrium, immune function or immunity can be divided into positive and negative parts, such as pro-inflammatory and anti-inflammatory factors (Cicchese et al., 2018), or anti-tumor and pro-tumor factors (Grivennikov et al., 2010), and so on. There are cytokines that promote the immune response (such as TNF-α and IFN-γ), and cytokines that inhibit the immune response (such as IL-10 and TGF-β) (Omer et al., 2003). There are positive immune cells, such as M1 macrophages, CD4^+^ helper T cells, and CD8^+^ effector T cells (Ahrends et al., 2019; Xing et al., 2021), and negative immune cells, such as M2 macrophages, regulatory T cells (Treg), myeloid-derived suppressor cells (MDSC) (Xing et al., 2021), and so on. While comparing all positive immunity (I_p_) to all negative immunity (I_n_), the immune equilibrium coefficient (C_ie_) can be obtained, i.e., C_ie_ = I_p_/I_n_, which represents the immune equilibrium index of a living organism as a whole. As mentioned above, I_p_ includes positive immune reserve (R_pi_) and positive immune power (P_pi_), that is, I_p_ = R_pi_ + P_pi_. Similarly, I_n_ also includes negative immune reserve (R_ni_) and negative immune power (P_ni_), that is, I_n_ = R_ni_ + P_ni_. If we temporarily ignore the immune reserve and only consider the immune power, that is, there are P_pi_ and P_ni_. In fact, P_pi_ is activated I_p_ and P_ni_ is activated I_n_. P_pi_ is composed of a series of positive dynamic factors, including positive immune factors and activated positive immune cells, which can be represented by X, i.e., X1, X2, X3 … Xn. In the same way, P_ni_ is composed of negative dynamic factors, including negative immune factors and activated negative immune cells, which can be represented by Y, i.e., Y1, Y2, Y3 … Yn. The ratio of positive and negative immune power is called the immune dynamic equilibrium coefficient (C_de_), which is the C_ie_ after removing the immune reserve. Thus, equation (1) as shown below is obtained, that is, the immune dynamic equilibrium equation. If the dynamic changes of C_de_ are plotted as a curve, this curve is called the immune dynamic equilibrium curve. Similarly, the P_pi_ (X1) (X2) (X3) … (Xn) and P_ni_ (Y1) (Y2) (Y3) … (Yn) also changes dynamically in the immune response, producing two curves, namely, the P_pi_ curve and P_ni_ curve. If we assume (whether this assumption is true or not, which will be discussed later) that the area under the curve (AUC) of the positive and negative immune power is equal throughout the entire immune response, namely, equation (2) as shown below, then we can do thought experiments and derive new concepts and new equations from these experiments.


(1) $$C_{de}=\frac{\left(X1\right)\;\left(X2\right)\;\left(X3\right)\ldots \left(\mathrm{Xn}\right)}{\left(Y1\right)\;\left(Y2\right)\;\left(Y3\right)\ldots \left(\mathrm{Yn}\right)}$$

(2) $$\frac{AUC\;\left(X1\right)\;\left(X2\right)\;\left(X3\right)\ldots \left(Xn\right)}{AUC\;\left(Y1\right)\;\left(Y2\right)\;\left(Y3\right)\ldots \left(Yn\right)}=1$$

**Figure 1:**
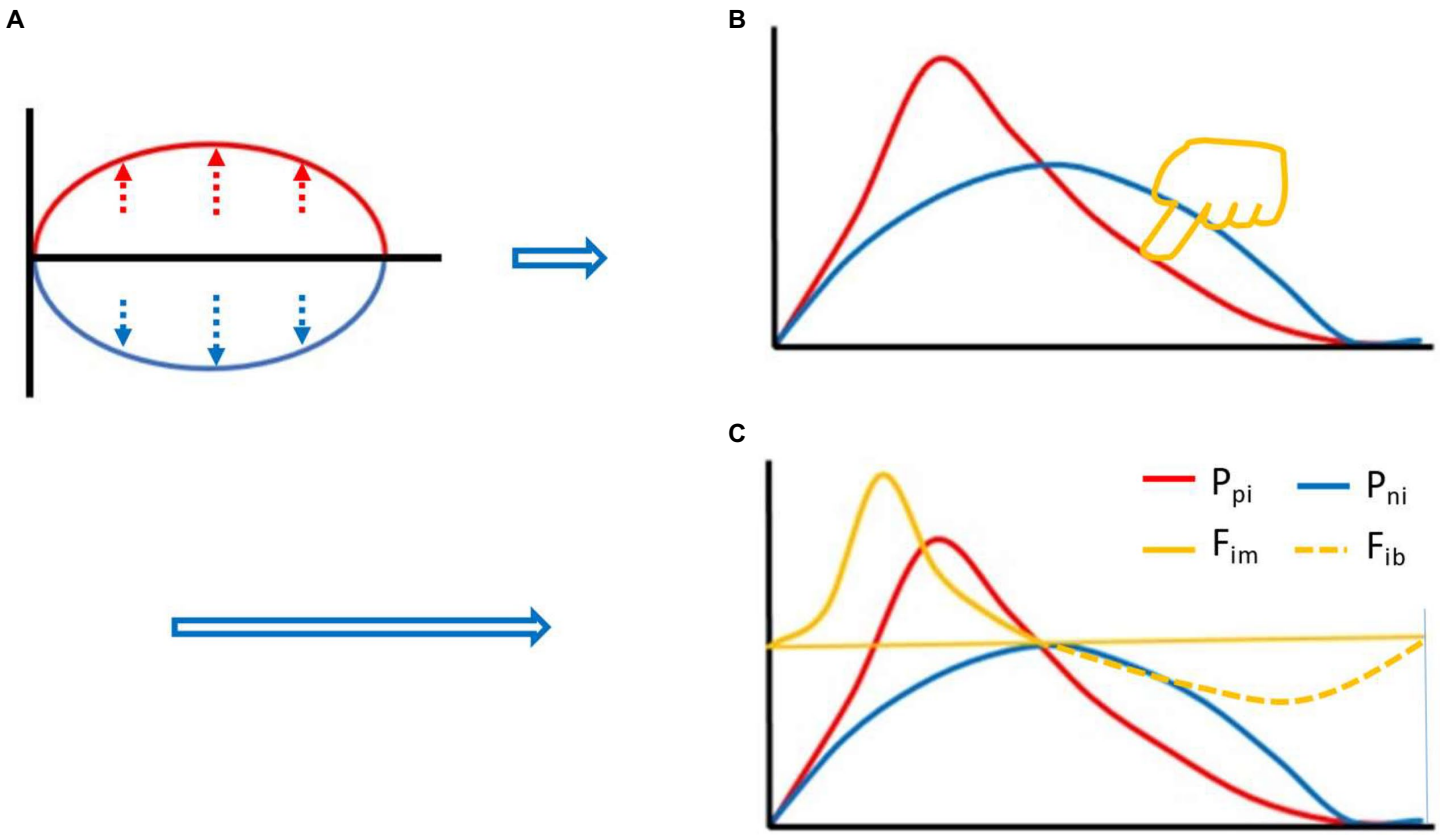
Thought experiment 1. It was assumed that during the whole process of immune response, the area under the curve (AUC) (red) of positive immune power (P_pi_) was equal to the AUC (blue) of negative immune power (P_ni_), namely AUC P_pi_ = AUC P_ni_, or equation (2) described in the main text. Before the immune response, both were at baseline, where the red and blue lines overlapped with the horizontal axis. Suppose the red line went up and the blue line went down, an oval with the same area above and below the horizontal axis was created **(A)**. Flipping the blue line up and down and overlapping the red line, and stretching both curves to the same extent. Then, pressing the red line from the upper right toward the lower left with appropriate force (finger in yellow color, **[B]**), an intersection point was created between the red line and the blue line (except for the starting and ending points) **(B)**. Before the intersection, the positive immune power was greater than the negative immune power, forming the immune force (F_im_) and its dynamic curve (**[C]**, yellow solid line), and obtaining its expression equation, namely, equation (3) described in the main text. After the intersection, the negative immune power was greater than the positive immune power, forming the immune braking force (F_ib_) and its dynamic curve (**[C]**, yellow dotted line), and obtaining its expression equation, namely, equation (4) described in the main text.

Based on equation (2), we can start with the two curves of Figure 1A. First of all, the red line represents the P_pi_, and the blue line represents the P_ni_. Before the immune response, both are at baseline, where the red and blue lines overlap on the horizontal axis. Suppose the red line goes up and the blue line goes down, and you get an oval with the same area above and below. However, if this was the case, the P_pi_ would be (completely) overcome by the P_ni_ at every instant, and an effective immune response would be impossible. In this case, if you flip the blue line up and down and overlap the red line, the area under the two curves is still the same. If you stretch both curves to the same extent, the AUCs are still the same. In this case, it can be assumed that we apply an appropriate amount of downward left force (yellow finger in Figure 1B) on the upper right side of the red line to deform the red line, but the AUC remains the same, the deformation curve (in red) shown in Figure 1B is obtained. The red line and the blue line form an intersection except for the starting point and the ending point, and the red line rapidly rises from the starting point, creating a difference in the rate of rise of the blue line. Before this intersection, the P_pi_ is greater than the P_ni_, their ratio, called the immune force (F_im_) and its relationship with the P_pi_ and P_ni_ is shown below in equation (3). Equation (3) is the F_im_ equation, which represents the force of P_pi_ overcoming the P_ni_ and the force to promote the immune response. The change of F_im_ value also forms a dynamic curve, called the F_im_ curve, which increases rapidly from the baseline level, decreases gradually after reaching the peak, and finally returns to the baseline level. The immune forces required for an effective immune response include the forces that drive both innate and adaptive immune responses. Here, we need to use immunological knowledge to describe the F_im_ curve: due to the rapid response of innate immunity, F_im_ will rise rapidly in the initiated stage dominated by innate immunity. However, the adaptive immune response is relatively slow and its process is longer (Marshall et al., 2018). Thus, the rapidly rising part of the curve mainly represents innate immunity, while the gradually declining part of the curve mainly represents adaptive immunity (although there is some overlap between the innate and adaptive immune responses), until an immune response is no longer required, such as the one induced by a pathogen infection, after the pathogen is cleared by the immune response, the body no longer needs an immune response, at this point, F_im_ falls back to baseline level. After this intersection, the P_ni_ is greater than the P_pi_, and the ratio between them is called immune braking force (F_ib_). The relationship among F_ib_ and P_pi_ and P_ni_ is shown below in equation (4), which is the immune braking force equation. It represents the force by which the P_ni_ overcomes the P_pi_ and immobilizes the immune response. This immune braking force would promote the formation of immune memory (see Discussion section). That is, the immune response is driven by the F_im_, and when the body does not need an immune response, the F_ib_ prompts the activated immune cells to gradually deactivate and become the resting memory cells. It is quite clear that the immune dynamic equilibrium curve is composed of the F_im_ curve and the F_ib_ curve, i.e., the yellow curve (solid line plus dotted line) in Figure 1C, which shows the relationship among the red, blue and yellow curves. It is important to note that the presence of a negative symbol “-” on the right side of equation (4) does not mean that the detected value of an immunodynamic factor is or could be negative. In practice, no test value is negative. This negative value in this equation is merely defined as the direction of the immune braking force is opposite to the direction of the immune force. It is also worth noting that if an immunodynamic factor is in the location of denominator in equation (3) or (4), and its detection value is zero, thus the equation would be invalid, this factor is then ineligible for inclusion in the practical immunodynamic equations which will be discussed later (an exception is made when the test method is not qualified).


(3) $$F_{im}=\frac{\left(X1\right)\;\left(X2\right)\;\left(X3\right)\ldots \left(Xn\right)}{\left(Y1\right)\;\left(Y2\right)\;\left(Y3\right)\ldots \left(Yn\right)}\;$$

(4) $$F_{ib}=-\frac{\left(Y1\right)\;\left(Y2\right)\;\left(Y3\right)\ldots \left(Yn\right)}{\left(X1\right)\;\left(X2\right)\;\left(X3\right)\ldots \left(Xn\right)}\;$$

**Figure 2:**
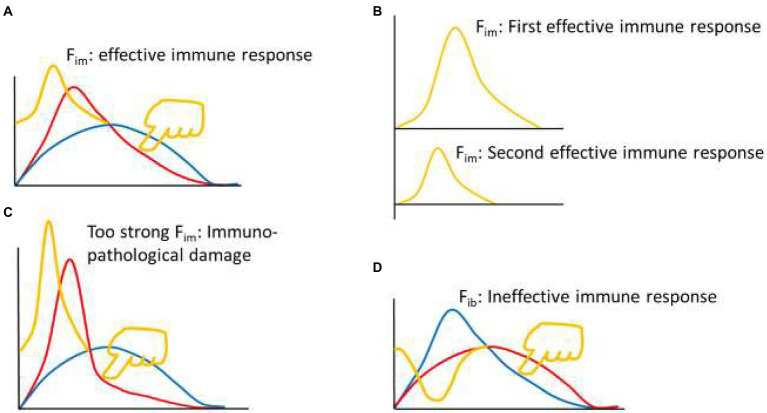
Thought experiment 2. **(A)** Pressing the red line from the upper right toward the lower left with appropriate force (finger in yellow color) to initiate appropriate F_im_ dynamics (yellow curve), produced an effective immune response. **(B)** From the perspective of F_im_, the second effective immune response was a smaller version of the first effective immune response. **(C)** Excessive pressure (finger in yellow color) to the red line induced excessive F_im_, leading to immunopathological damage. **(D)** Pressing the blue line from the upper right toward the lower left (finger in yellow color) induced dynamic changes of immune braking force (F_ib_, yellow curve) and produced an ineffective immune response.

The reason there is an immune braking force (F_ib_) process after immune force (F_im_) is that immune braking is an active braking process like car braking, rather than a passive braking process like friction between wheels and road surface. For example, effector immune cells often express costimulatory molecules (such as OX-40, CD40L, GITR, and 4-1BB) and coinhibitory molecules (such as PD-1, CTLA-4, TIM-3 and LAG3) simultaneously during activation (Viganò et al., 2012; Pan et al., 2021), but the ratio between the two sets of molecules is constantly changing during the immune response. An appropriate range of F_im_ dynamics represents an effective immune response (yellow curve in Figures 2, A and B up). The F_im_ dynamics of a re-reaction of an effective immune response (as in the case of a pathogen reinfection) is a miniaturized version of the first immune response (Figure 2B down). A rapid and substantially increased dynamic change in F_im_ represents an overactive immune response (yellow curve in Figure 2C), usually resulting in immunopathological damage or severe immunopathological damage. A response that initiates a process equivalent to dynamic F_ib_ is an ineffective immune response (yellow curve in Figure 2D). These equations are called theoretical equations of immunodynamics, these curves are called theoretical curves of immunodynamics, and their synthesis is the theoretical model of immunodynamics.

### A practical model of immunodynamics

In mathematics, the equivalent transformation of equation (3) can be carried out, which is called factor pair equivalent transformation, or factor pair transformation for short. In other words, the positive dynamic factors and the negative dynamic factors are paired one by one to make them into factor pairs. Of course, this pairing is based on existing immunological knowledge, such as TNF-α/IL-10, IFN-γ/TGF-β, etc., hence equation (5) is obtained:


(5) $$F_{im}=\left(\frac{X1}{Y1}\right)\left(\frac{X2}{Y2}\right)\left(\frac{X3}{Y3}\right)\ldots \left(\frac{Xn}{Yn}\right)$$

Equation (5) has an essential practical significance, that is, data from different laboratories or different experiments in the same laboratory can be combined and used, as long as the following two requirements are met, it is completely in line with mathematical logic. In this way, each studied factor pair can be found in the published literatures or databases, and their values can be substituted into equation (5) to obtain the F_im_ value. Starting with a factor pair, add factor pairs one by one, then calculate the F_im_ values, and draw a graph according to the F_im_ values to get the dynamic curve of F_im_. Then, these curves are compared and fitted with the theoretical F_im_ curve mentioned above, and the curve with a high degree of fitting may be found. Finally, the approximate equations of immunodynamics, namely, the practical equations of immunodynamics, can be found through these curves. However, in the process of looking for the immunodynamic practical equations, I found that in front of an immune response, the F_im_ baselines were usually different. To make the baselines to become equal, and to give the concept of F_im_ an intuitive thinking which is easy to be understood, we can assume that in front of an immune response, the positive and negative immune powers are equal, and their ratio is equal to 1. This can be done mathematically, as long as an adjustment coefficient (C_oe_) is introduced into equation (5), and C_oe_ = [(Y1) (Y2) (Y3) … (Yn)]/[(X1) (X2) (X3) … (Xn)]. Mathematically, C_oe_ can ensure that the F_im_ value before an immune response is at the baseline level of 1, so the F_im_ equation (6) is obtained:


(6) $$\;F_{im}=\left(\frac{X1}{Y1}\right)\left(\frac{X2}{Y2}\right)\left(\frac{X3}{Y3}\right)\ldots \left(\frac{Xn}{Yn}\right)\left(C_{oe}\right)$$

Thus, published data on factor pairs (e.g., TNF-α/IL-10, IFN-γ/TGF-β, etc.) can be substituted into equation (6) to calculate F_im_. As mentioned earlier, two basic conditions must be met to perform such an operation. First, the two factors of a factor pair must come from the same experiment and have the same units. Second, different factor pairs can come from different experiments, but they must belong to the same experimental system. For example, a laboratory conducted a study on the infection of the nonlethal strain of *Plasmodium yoelii* (Py) in C57BL/6 mice and observed the dynamic changes of plasma TNF-α/IL-10 levels induced by *Plasmodium* infection. The detection method was ELISA, and both TNF-α and IL-10 were measured in ng units. In another laboratory, the nonlethal strain of Py was also used to infect C57BL/6 mice, and the changes of plasma IFN-γ and TGF-β levels induced by the infection were observed. The detection method could be ELISA or other methods, and the units of IFN-γ and TGF-β were both μg. At the same time, the result of parasite infection must be basically the same, for example, C57BL/6 mice infected with nonlethal Py did not die, the *Plasmodium* parasites disappeared in the same or similar time, and the time point of observation of the immune factor pairs was the same or roughly the same, etc., to judge from the professional, because the rigor of mathematical logic must be based on a logical system. If these conditions are met, then the two pairs of factors from the two laboratories can be combined. To take a reverse example, if *Plasmodium berghei* (Pb) was used in the second laboratory, even though the mice used were still C57BL/6 mice, regardless of the units measured for IFN-γ and TGF-β, regardless of whether the detection method was the same, it could not be combined with the data from the first laboratory. This is not only the mathematical logic requirement of equation (6), but also the requirement of specialized knowledge in immunology, because Pb is lethal to C57BL/6 mice, while nonlethal strain of Py is nonlethal to C57BL/6 mice.

**Figure 3:**
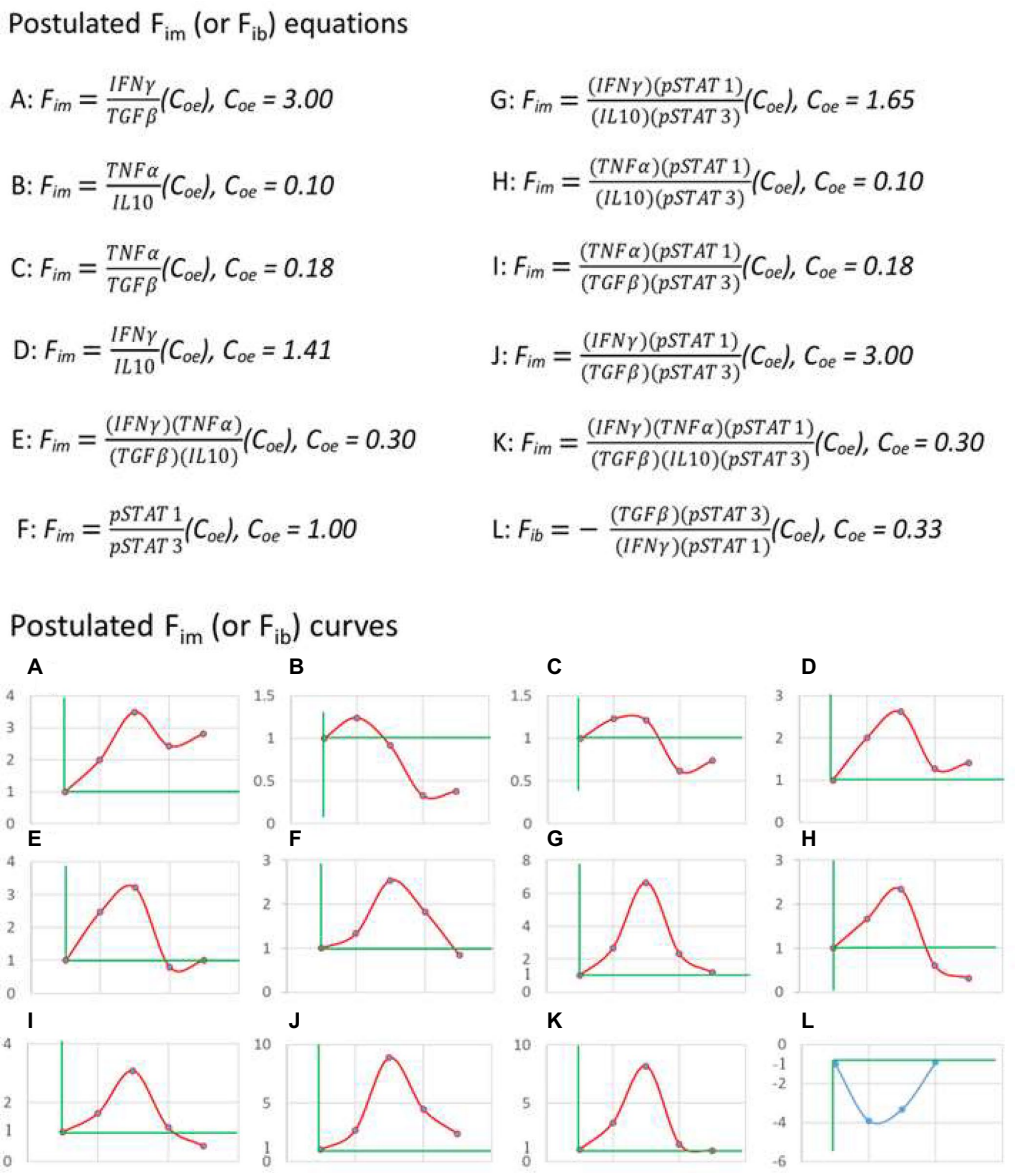
The practical immunodynamic equations were screened by the practical calculation of functional data. The process of obtaining some practical equations of immunodynamics based on the data (IFN-γ, TGF-β, TNF-α, and IL-10) of Reference (Omer et al., 2003) and the data (pSTAT1 and pSTAT3) of Reference (Shi et al., 2008). From the postulated equations (up panel) to their corresponding curves (down panel), and through the comparison and fitting of these curves with the theoretical curve shown in Figure 2B, the fitted and deduced curves (J and L, down panel) were discovered and their corresponding equations (J and L, up panel) were selected. The data used in A-K came from C57BL/6 mice infected with non-lethal Py, and the data used in L came from C57BL/6 mice infected with lethal Py. Curves A-K: The first dot of the curve represented the baseline level (F_im_ = 1) before *Plasmodium* infection (on day 0), the second dot represented (the data on) day 1 after infection, the third dot represented day 7, the fourth dot represented day 14, the fifth dot represented day 21; data on day 16 in reference (Omer et al., 2003) were combined with data on day 14 in reference (Shi et al., 2008) as the data on day 14, and data on day 20 in reference (Omer et al., 2003) were combined with data on day 21 in reference (Shi et al., 2008) as the data on day 21. Curve L: The first dot represented the baseline level (F_ib_ = −1) before infection (on day 0), the second dot represented day 1 after infection, the third dot represented day 3, the fourth dot represented day 7; data on day 2 or day 3 in Reference (Omer et al., 2003) were combined with data on day 3 in Reference (Shi et al., 2008) as the data on day 3. Someone may ask, what values reign over C_oe_ value in an equation, or a time curve? The answer is that the actual detected values at baseline determine the C_oe_ value. For example, in up panel A, why C_oe_ = 3? The answer is that, based on the data of Literature A (Omer et al., 2003), at baseline (before an immune response), IFNγ = 2 (μg/ml), and TGFβ = 6 (μg/ml), thus, C_oe_ = 6/2 = 3 (C_oe_ = Y/X). This can ensure that F_im_ = (2/6) (3) = 1 at baseline.

Based on the above principles, I began to search for a practical equation of immunodynamics. For example, some papers were selected from the PubMed database in which the authors infected C57BL/6 mice with the nonlethal strain of Py for observing the dynamics of immune factors. Firstly, the data of paper (Omer et al., 2003) from Laboratory A were used to calculate the dynamic changes of plasma IFN-γ, TGF-β, TNF-α, and IL-10 levels in mice induced by *Plasmodium* infection. Starting with two-factor (one pair) equation (Figure 3, up panel A, B, C, and D), that was, assuming F_im_ = (X1/Y1)(C_oe_), the data of the factor pairs were substituted into the equations, and the resulting F_im_ values were used to compose the graphs (Figure 3, down panel A, B, C, and D). It was found that these curves did not fit the theoretical curve in Figure 2B. Then the four-factor (two-pair) equation (Figure 3, up panel E) was used, namely, it was assumed that F_im_ = (X1/Y1)(X2/Y2)(C_oe_), and the data of Laboratory A were also used. It was found that, like the two-factor equations, the dynamic curve (Figure 3, down panel E) generated based on the four-factor equation did not fit the curve in Figure 2B. Then, the literature (Shi et al., 2008) published by Laboratory B (my laboratory) was used. The authors of this paper also infected C57BL/6 mice with the nonlethal strain of Py and observed the dynamic changes of pSTAT1 and pSTAT3 in the spleen immune cells of mice. When pSTAT1/pSTAT3 were used as the dynamic factor pair to substitute into the two-factor equation (Figure 3, up panel F), it was found that the dynamic curve (Figure 3, down panel F) could not fit the curve in Figure 2B. And then, all factors of Laboratory A and Laboratory B were combined and used, because the two laboratories used the same experiment system, and such a combination met the requirements of mathematical logic and professional knowledge mentioned above. For example, both of them used nonlethal Py and C57BL/6 mice, with very similar results that the parasite disappeared from the body on average 28 days, the mice did not die, and the observation time points of the immune factors were also very similar. Through different combinations of factor pairs, I tested the four-factor equations (Figure 3, up panel G, H, I, and J) and obtained their corresponding curves (Figure 3, down panel G, H, I, and J) according to the equations. It was found that the curve (Figure 3, down panel J) produced by equation J, one of the four-factor equations with combined data from two laboratories, fitted the theoretical curve (Figure 2B). Then, based on the four-factor equation, the data of other factor pairs from the two experimental bodies were introduced to establish the six-factor equation (Figure 3, up panel K), and it was found that the curve (Figure 3, down panel K) generated by the six-factor equation did not fit the theoretical curve. Therefore, it can be preliminarily determined that equation (7) as shown below is a practical F_im_ equation, namely, one of the practical equations of immunodynamics.


(7) $$F_{im}=\frac{\left(IFN\gamma \right)\;\left(pSTAT1\right)}{\left(TGF\beta \right)\;\left(pSTAT3\right)}\left(C_{oe}\right)$$

In mathematical logic, the reverse is also true. For example, using equation (7) and the above two-laboratory data, the dynamic curve (Figure 3, down panel J) of F_im_ values for C57BL/6 mice infected with nonlethal Py could be obtained. This curve showed that the F_im_ value from the baseline level (1) began to rise rapidly, within 7 days to reach the peak. This should be equivalent to the immune response process dominated by innate immunity, and then to gradually decline relatively slowly. On day 21, it was still higher than the baseline level, because the malaria parasite had not been cleared. In theory, when the parasite is completely cleared after 28 days (mean), the F_im_ level would drop to the baseline level. The decline process of F_im_ mainly corresponds to the adaptive immune response phase. This dynamic process of F_im_ is highly consistent with existing immunological knowledge.

Then we could use equation (7) to investigate the immune dynamics induced by various immune interventions. For instance, we could inspect the results of C57BL/6 mice infected with lethal Py, this would be another example to verify the validity of equation (7). The lethal and nonlethal strains of Py are two different strains of the same species. The former would cause all C57BL/6 mice to die within 7 days after infection, while the latter would cause no death of C57BL/6 mice. That means, the mice have ability to completely clear the nonlethal parasite in about 28 days and then completely recover. The data (Omer et al., 2003) from Laboratory A and the data (Shi et al., 2008) from Laboratory B were also used, and the experimental results of C57BL/6 mice infected with lethal Py were reported, respectively. Both laboratories observed that all C57BL/6 mice died within 7 days after being infected with lethal Py. The data integration of the two experiments fully complied with the above mathematical logic and the requirements of related majors. In the same way as above, the relevant data were introduced into equation (7), and it was found that after *Plasmodium* infection, the F_im_ value always changed below 1 until all mice died. As mentioned above, this situation was equivalent to the dynamic change of immune braking force. Therefore, according to equations (4) and (7), equation (8) as shown below could be obtained. That means, equation (8) is the practical immune braking force equation, another one of the practical equations of immunodynamics.


(8) $$F_{ib}=-\frac{\left(TGF\beta \right)\;\left(pSTAT3\right)}{\left(IFN\gamma \right)\;\left(pSTAT1\right)}\left(C_{oe}\right)$$

Vice versa, the relevant data of the above two literatures were substituted into equation (8) (Figure 3, up panel L) to obtain the immune braking force curve (Figure 3, panel L). This curve indicated that the lethal Py induced immune braking force rather than immune force at the early stage of infection, and thus was unable to induce an effective immune response. Therefore, the malaria parasite rapidly reproduced, resulting in the death of all infected mice within 7 days. These results suggest that equations (7) and (8) can be regarded as a practical immunodynamic equation set preliminarily verified by positive and negative functional data.

It should be noted that equation (2) is artificial and assumed only for thought experiments and mathematical derivation. For example, the concepts of immune force F_im_ and immune braking force F_ib_ and their calculation formulae are derived from this derivation, and they have been proved to be real by actual calculation (through functional data of C57BL/6 mice infected with nonlethal and lethal Py). However, after obtaining these new concepts and their expression formulae, equation (2) must be forgotten, because it is sufficient to derive F_im_ and F_ib_ and their expressions. It is not necessary to prove AUC F_im_ = AUC F_ib_ or AUC F_im_ ≠ AUC F_ib_, because this has no practical significance at least at the present stage.

From the perspective of immunodynamics, we can redefine the concept of immune equilibrium as that the immune system maintains or returns to the pre-reaction state of homeostasis through the mutual restriction between the immune force and immune braking force. According to this definition, the immune response can be classified as equilibrium and disequilibrium, which can be further divided into immune force-dominated and immune braking force-dominated, and acute or chronic. For example, the immune response of C57BL/6 mice infected with nonlethal Py belongs to the acute equilibrium. The immune response of C57BL/6 mice infected with lethal Py belongs to the immune braking force-dominated acute disequilibrium, that is, the reaction process is characterized by only a temporary immune braking force. Similarly, after a long-term immunoediting process (Dunn et al., 2004; O'Donnell et al., 2019), the immune system of cancer patients (especially advanced cancer patients) is in a state of immune braking force-dominated chronic disequilibrium, that is, the immune process dominated by immune braking force for a long period. This disequilibrium is more obvious in tumor tissues than in peripheral blood. In some autoimmune diseases, such as rheumatoid arthritis and multiple sclerosis (Annunziato et al., 2015), the immune system is in a state of immune force-dominated chronic disequilibrium.

At the same time, it should be emphasized that the above theoretical equations of immunodynamics, namely, equations (3)–(6), could be applicable to all types of cellular (and even humoral) immune responses, but the practical equations of immunodynamics proposed in this paper, namely equations (7) and (8), are only applicable to type 1 cell-mediated immune response. This is because the data used to derive the generation of equations (7) and (8) are only from C57BL/6 mice infected with *Plasmodium* parasite, an intracellular pathogen whose infection induces a type 1 cell-mediated immune response, like those induced by infections with viruses and intracellular parasitic bacteria (Annunziato et al., 2015). Tumor immunity is also mainly a type 1 cell-mediated immune response (Klinke, 2014; Galon and Bruni, 2020), and many autoimmune diseases are caused by type 1 immune disequilibrium, such as insulin-dependent (type 1) diabetes mellitus, inflammatory bowel disease, autoimmune gastritis, Hashimoto thyroiditis, and the aforementioned rheumatoid arthritis and multiple sclerosis (Annunziato et al., 2015). Therefore, equations (7) and (8) can be used to study the immunodynamic process of intracellular pathogen infection, tumor, and the above-mentioned autoimmune diseases. Research scientists who are engaged in the study that is related to type 2, type 3 cellular, and even humoral (highly associated with type 2 cell-mediated) immune responses can find practical equations of immunodynamics in their respective fields according to the methods presented in this paper (the author of this paper is only engaged in the study that is related to type 1 cellular immune responses).

When applying equations (7) and (8), it should also be noted that if they (F_im_ and F_ib_) are used to study the process of a disease, it is sometimes impossible to obtain the baseline data before the disease occurs. In this case, the data of healthy people can be used as the baseline level. For example, to observe the dynamic changes in F_ib_ of peripheral blood of cancer patients, the F_ib_ value of peripheral blood of normal people can be used as the baseline level, which must be 1 (because C_oe_ is included in the equation), and then the F_ib_ value of cancer patients can be calculated and the dynamic changes can be observed. However, if immunotherapy is given to cancer patients, dynamic changes in F_im_ values should be observed. Before treatment, the baseline F_im_ level of patients must be 1 (because C_oe_ is in the equation), and then dynamic changes in F_im_ values during immunotherapy should be observed. To be effective in cancer immunotherapy, F_im_-dominated immune responses must be induced. If F_ib_-dominated immune responses are induced, they may lead to hyperprogression of disease (Han et al., 2020) (discussed later). On the contrary, for the immunotherapy of autoimmune diseases caused by the chronic disequilibrium of the type 1 cellular immune response, it is necessary to induce the immune response dominated by F_ib_ to be effective. Since the dynamic changes of F_im_ and F_ib_ values are individualized and can be used as biomarkers, the practical equations of immunodynamics can be used to guide individualized immunotherapy.

### Energy-based immunodynamics

All above equations for establishing immunodynamics are from the point of view of substances, including their underlying information. To further understand the nature of immunodynamics and its profound significance, it is necessary to explain immunodynamics from the perspective of energy, so the concept of net immune energy (immune energy for short, E_im_) needs to be introduced. Based on Einstein’s mass-energy relation equation E = mc^2^,^1^ all the mass of matter can be converted into energy. Thus, if a substance representing P_pi_ (including its implied information) releases its energy to drive the immune response, this energy can be defined as positive immune energy (E_pi_); in the same way, if a substance representing P_ni_ (including its implied information) releases its energy to block the immune response, this energy can be defined as negative immune energy (E_ni_). According to the substance-based equation F_im_ = P_pi_/P_ni_, the energy-based theoretical immunodynamic equation, namely, the theoretical immune energy equation (9), could be obtained (shown as below). As is known to all, adenosine triphosphate (ATP) acts as a unit of energy in living organisms, and based on the studies of immunometabolism, it is believed that extracellular ATP (eATP) promotes the immune responses, while extracellular adenosine (eADO), the metabolite of ATP, inhibits immune responses (Takenaka et al., 2016; Faas et al., 2017; Dwyer et al., 2020). Therefore, the ratio of eATP/eADO can be used to construct the energy-based approximate immunodynamic equation, namely, the approximate immune energy equation (10) (shown as below). It is important to note that in mathematical logic, the same thing with the same unit of measurement in the equation can be calculated with simple addition, not necessarily with multiplication, although multiplication is not impossible, such as the situation in the energy-based equation (10), while different things (even the same thing) with different units of measurement can only be calculated with multiplication but not addition, such as the situations in the substance-based equations. Nevertheless, these immune energy equations only help us to understand the nature of immunodynamics and their profound implications. They are not yet applicable to the field of immunology today, and may be applied in the future after further development in the study of immunometabolism, an important branch of immunology (O'Neill et al., 2016; Jung et al., 2019; Pearce, 2021). At a minimum, equation (10) needs to be verified by functional data, such as eATP/eADO data of C57BL/6 mice infected with nonlethal and lethal Py, which conform to the dynamic change processes shown in Figures 2B,D, 3 down panel J and L, respectively, before it can be considered as a practical immune energy equation. As it is not yet in use, there is no need to introduce the adjustment coefficient (C_oe_) into this equation, and no need to convert it to a reciprocal form at the present stage.


(9) $$E_{im}=E_{pi}/E_{ni}$$

(10) $$E_{im}=\sum eATP/\sum eADO$$

**Figure 4:**
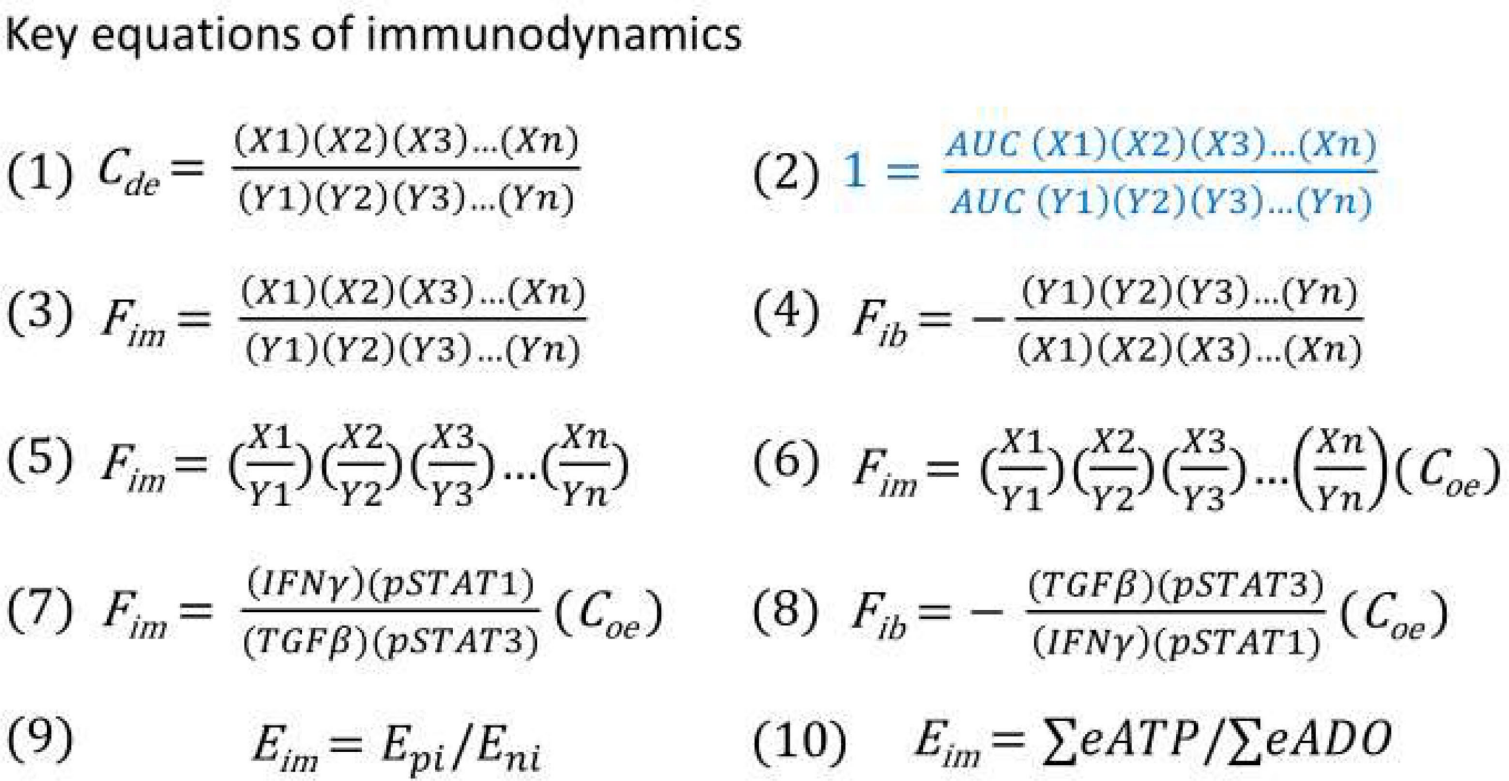
(1) Immune dynamic equilibrium equation. (2) The equation for thought experiments based on the postulate (regardless of correct or not) that the AUC of the positive and negative immune power was equal throughout the entire immune response. (3) Theoretical immune force equation when positive immune power > negative immune power. (4) Theoretical immune braking force equation when negative immune power > positive immune power. (5) Immune force equation from equation (3) after conducting factor pair equivalent transformation. (6) Immune force equation obtained from equation (5) after the adjustment coefficient (C_oe_) was introduced for ensuring that the F_im_ value before an immune response was at the baseline level of 1. (7) Practical Immune force equation obtained based on equation (6) through calculation of functional data. (8) Practical immune braking force equation based on equations (4) and (7) through calculation of functional data. (9) Theoretical energy-based immunodynamic equation. (10) Approximate energy-based immunodynamic equation that has not yet been verified by functional data.

Since a serial of new concepts and equations are proposed for constructing the model of immunodynamics, it is necessary to summarize them in Table 1 and Figure 4, respectively.

**Table 1:** Key concepts of immunodynamics.

Concept	Symbol and definition	Significance
Positive immunity	I_p_ = R_pi_ + P_pi_	Positive part of the immune system
Negative immunity	I_n_ = R_ni_ + P_ni_	Negative part of the immune system
Immune equilibrium coefficient	C_ie_ = I_p_/I_n_	Immune equilibrium index of a living organism as a whole
Positive immune reserve	R_pi_ = I_p_ − P_pi_	Resting part of positive immunity
Negative immune reserve	R_ni_ = I_n_ − P_ni_	Resting part of negative immunity
Positive immune power	P_pi_ = I_p_ − R_pi_	Activated part of positive immunity
Negative immune power	P_ni_ = I_n_ − R_ni_	Activated part of negative immunity
Immune dynamic equilibrium coefficient	C_de_ = P_pi_/P_ni_	The C_ie_ after removing the immune reserve
Immune force	F_im_ = P_pi_/P_ni_, when P_pi_ > P_ni_	The force of positive immune power overcoming the negative immune power and promoting the immune response
Immune braking force	F_ib_ = P_ni_/P_pi_, when P_ni_ > P_pi_	The force by which the negative immune power overcomes the positive immune power and immobilizes the immune response
Immune factor pair	Positive factor/negative factor	A factor pair must come from the same experiment and have the same units; different factor pairs can come from different experiments, but they must belong to the same experimental system
Adjustment coefficient	C_oe_ = P_ni_/P_pi_ at baseline	C_oe_ can ensure that the F_im_ value before an immune response is at the baseline level of 1
Immune energy	E_im_ = E_pi_/E_ni_	Net immune energy that has overcome immune resistance to drive immune response
Positive immune energy	E_pi_ = (E_im,_) (E_ni_)	The part of immune energy to promote immune response
Negative immune energy	E_ni_ = E_pi_/E_im_	The part of immune energy to inhibit immune response

### Prediction and application of immunodynamic theory

#### Individualized treatment for COVID-19 patients

The world is currently in the midst of the COVID-19 pandemic. As a newly emerging viral infectious disease, the immune reaction induced by the infection of its pathogen (SARS-CoV-2) (Yuki et al., 2020) must also belong to the type 1 cell-mediated immune response. Therefore, equations (7) and (8) can be used to describe its immunodynamic process. Based on the analysis of existing literature (Leisman et al., 2020; Yuki et al., 2020; Olbei et al., 2021), the following immunodynamic predictions are made. First, the majority of patients, namely, asymptomatic infected persons and mild to moderate infected persons, should have an acute equilibrium type of immune response, namely, an effective acute immune response is generated (Mai et al., 2021; Schiffner et al., 2021). In the absence of specific antiviral drug treatment, the immune system itself can eventually overcome the virus and the patients recover completely. Second, some patients’ immune response belongs to the acute immune equilibrium at the beginning, but about a week after the clinical symptoms, suddenly turns into F_im_-dominated acute disequilibrium, resulting in an overactive immune response and developing severe or critically ill. In other words, the F_im_ value should have started to decline during the transformation from innate to adaptive immunity, but the F_im_ value of these patients does not drop and continues to rise, resulting in higher F_im_ dynamics and longer duration. In addition to other treatment measures, these patients need to use corticosteroids or other immunosuppressive agents to successfully cure the disease. Another group of patients experience acute immune equilibrium at the beginning, and then quickly turn into acute immune disequilibrium dominated by immune braking force, so that they are unable to produce effective immune responses, leading to severe or critically ill situations. These patients cannot use corticosteroids or immunosuppressive agents, instead, they need to use immune stimulators such as IFN-γ, but not IFNα and IFNβ, which may be more suitable for controlling viral replication in the early stages of infection (Channappanavar et al., 2019; Alavi Darazam et al., 2021) and have a limited role in immunodynamics. Therefore, the dynamic measurements of the immunodynamic parameters of each COVID-19 patient can be used to guide the individualized treatment. The immunodynamic parameters can be detected simply twice a week after onset (if there is baseline data before onset, it is the best; if there is no baseline data, it is necessary to use normal people’s data as the baseline). One milliliter of peripheral blood is taken at each time, then plasma and peripheral blood mononuclear cells (PBMC) are separated. Plasma is used to detect IFN-γ and TGF-β (Omer et al., 2003). PBMC is used to detect pSTAT1 and pSTAT3 (Shi et al., 2008). The F_im_ value is calculated according to equation (7). In the acute phase of infection, when the F_im_ value fluctuates within the range which is appropriately higher than 1, it should belong to the acute immune equilibrium. If the F_im_ value fluctuates at a much higher level above 1, it should belong to the acute disequilibrium dominated by the F_im_. If the F_im_ value fluctuates below 1, it belongs to the acute disequilibrium dominated by the F_ib_. Then, equation (8) should be used to calculate F_ib_ value to observe its dynamics, which will help clinical doctors to select and determine the dose and course of use of immune stimulators. It is suggested that hospitals with COVID-19 patients carry out immunodynamic studies as soon as possible, to quickly implement individualized treatment plans for COVID-19 patients and save more severe and critically ill patients.

#### Individualized programs for cancer immunotherapy

There are two major breakthroughs in cancer immunotherapy in the recent years (Couzin-Frankel, 2013), one of these is chimeric antigen receptor (CAR) T cell therapy, which has been shown to be very effective in the treatment of hematologic tumors, but limited in the treatment of solid tumors (June et al., 2018; Titov et al., 2020). CAR T cells kill some tumor cells, then the dead tumor cells release damage-associated molecular patterns (DAMPs) and tumor antigens, which induce endogenous innate and adaptive antitumor immune responses (Hernandez et al., 2016). As mentioned above, tumor immunity is mainly a type 1 cellular immune response, so theoretically, the effectiveness of CAR T cell therapy can also be determined by equations (7) and (8). Blood tumors have no fixed cancer nests, so the effectiveness can be determined by the dynamic changes of F_im_ values in peripheral blood. Moreover, the course of treatment can be determined according to the changes of F_im_, and the dosage of treatment can also be adjusted according to the F_im_ dynamics. At the same time, F_im_ in peripheral blood can also be used to predict the occurrence of cytokine release syndrome (CRS), a severe side effect of CAR T cell therapy and other immunotherapies (Shimabukuro-Vornhagen et al., 2018). Based on the published data (such as high level of IFN-γ) (Shimabukuro-Vornhagen et al., 2018; Cosenza et al., 2021), CRS can be judged as type 1 acute disequilibrium dominated by F_im_, therefore equation (7) can be used while combined with IL-6 level (one of the characteristics of CRS) for a comprehensive judgment. High level of IL-6 contributes to many of the key complications of CRS, such as vascular leakage, activation of complement and coagulation cascade inducing disseminated intravascular coagulation (DIC), therefore, once confirmed as the CRS, monoclonal antibodies against IL-6 and its receptor should be used (Shimabukuro-Vornhagen et al., 2018; Cosenza et al., 2021), and F_im_ value in combination with IL-6 data can be used to determine the dose and course of drugs required. Another breakthrough is the use of immune checkpoint inhibitors that have been shown to be effective in the treatment of a variety of solid tumors (Hargadon et al., 2018; Wei et al., 2018; Waldman et al., 2020). To judge whether these inhibitors are effective against solid tumors, immunodynamic parameters in peripheral blood and tumor tissue are needed. If there is an immune response dominated by F_im_ in tumor tissue, it should be judged as effective. Peripheral blood data can also be used for evaluation if intratumor immunodynamic data are not available. At the same time, immunodynamic parameters can also be used to evaluate the severe adverse reactions caused by checkpoint inhibitors, including CRS. If the checkpoint blockade therapy induces an acute disequilibrium dominated by immune braking force, I predict that this response would lead to hyperprogression of disease (Champiat et al., 2018; Han et al., 2020; Lin et al., 2021). If this happens, treatment should be discontinued immediately and other therapies should be used depending on the situation. More importantly, treatment with an immune checkpoint inhibitor should be terminated immediately if it induces a F_ib_-dominated ineffective immune response, rather than waiting for the occurrence of hyperprogression. Therefore, immunodynamic parameters can not only be used as biomarkers in cancer immunotherapy to judge the efficacy, but can also be used to formulate individualized treatment plans, determine the dose and course of treatment, and can be used to judge serious toxic and side effects and guide the prevention and treatment of these adverse events.

#### Anticancer mechanism of *Plasmodium* immunotherapy and its individualized approaches

The author of this paper and colleagues have long been engaged in the research of *Plasmodium* infection against cancer. Through murine model studies, we have demonstrated that *Plasmodium* infection activates the host immune system, induces antitumor innate and adaptive immune responses (Chen et al., 2011; Liu et al., 2017; Pan et al., 2021), relieves tumor immunosuppressive microenvironment (Adah et al., 2019), inhibits tumor angiogenesis through a series of mechanisms (Yang et al., 2017; Qin et al., 2020; Wang et al., 2020), inhibits cancer epithelial mesenchymal transformation (EMT) (Liang et al., 2021), and inhibits tumor growth and metastasis, therefore, significantly prolongs the life of tumor-bearing animals (Chen et al., 2021). Based on our research, clinical trials of *Plasmodium* immunotherapy for advanced cancer have been approved and are ongoing in China (NCT02786589, NCT03474822, and NCT03375983). We recently propose the notion that cancer is an ecological disease and *Plasmodium* immunotherapy is a systematic ecological counterattack therapy, and preliminarily describe the clinical safety and public health security of this therapy (Chen et al., 2021). Interestingly, our recent study indicates that subsequent *Plasmodium* infection induces a high proportion of CD4^+^ CD28^high^ CD95^high^ central memory T cells and a strong SIV (simian immunodeficiency virus)-specific T cell response, drives the hosts to maintain the diversity of SIV-specific T cell receptor repertoire, to generate new SIV-specific T cell clones to track the antigenic variations of SIV, and thus extends the life span of rhesus monkeys infected with SIV (Liu et al., 2022). This suggests that *Plasmodium* infection may also drive T cells in patients with cancer to trace the antigenic variations of cancer cells, thus potentially overcoming drug resistance and recurrence of tumor. If we examine the mechanisms of action of *Plasmodium* immunotherapy against cancer from the angle of immunodynamics, the immune system of people with cancer as dominated by F_ib_ (chronic type 1 immune disequilibrium), an acute and subacute infection of a benign form of malaria parasites (such as *Plasmodium vivax*) dominated by F_im_ is just able to confront this immune imbalance induced by cancer. The anticancer mechanism of other intracellular parasitic pathogens (such as BCG, oncolytic virus, etc.) (Kaufman et al., 2015; Pettenati and Ingersoll, 2018) should be very similar, but the intensity and duration of the induced F_im_ are different. However, in some situations, pathogen infection may also induce immune braking force, for example, lethal Py infection in C57BL/6 mice. Therefore, we are planning to monitor the immunodynamic parameters of each cancer patient in the next clinical trials to guide the individualized treatment regimen of *Plasmodium* immunotherapy. For example, the doctors currently conducting the clinical trials do not know exactly when is the beginning of *Plasmodium* immunotherapy, namely, at the time of parasite inoculation or at the time when the parasites can be detected in peripheral blood? But it is now clear that the time to start is when F_im_ >1. At the same time, from theory to practice, the doctors also do not know when should end the treatment, but in terms of immunodynamics, if treatment induces a F_im_-dominated immune response, the dynamic change of F_im_ value should be in line with the dynamic curve in Figure 2B, and when the F_im_ value falls back to the level that is slightly higher than 1, the course of treatment should be finished, instead of the current 4–8 weeks of treatment course. In addition, if *Plasmodium* immunotherapy induces a F_ib_-dominated ineffective immune response in some patients, treatment should be discontinued immediately, rather than waiting for significant tumor progression as currently observed in clinical trials. At the end of treatment, the AUC of F_im_ (AUC F_im_) can be used to determine the strength and duration of the immune response and to predict the treatment efficacy. In this way, *Plasmodium* immunotherapy will become a highly individualized treatment through the application of immunodynamics. This approach could also be used in other immunotherapies.

## Discussion

Modern immunology has become very complicated, involving complex theories and advanced technologies such as genomics (Neu et al., 2017), transcriptomics (Stubbington et al., 2017) and metabolomics (Everts, 2018). However, its core principle seems to be only the immune recognition theory (del Guercio, 1993; Janeway and Medzhitov, 2002; Kubelkova and Macela, 2019; Minervina et al., 2019; Joyce and Ternette, 2021; Werner et al., 2021), and the immune equilibrium theory (Swiatczak, 2014; Eberl, 2016; Cicchese et al., 2018; Shevyrev et al., 2021) is limited to the analysis of the philosophical significance of immune function. In this paper, I attempt to find an immunological theory like the simple laws of physics, and put forward the theoretical framework of immunodynamics to start the research in this novel field. First, by mathematizing the traditional concept of immune equilibrium, the equation (1) is obtained. Then, a key postulate is proposed, that is, the AUC of the P_pi_ is equal to the AUC of the P_ni_, namely the AUC P_pi_ = AUC P_ni_ [equation (2)]. Based on this postulate, a series of thought experiments have been conducted, such as, through the change of one curve, the two curves create an intersection point, before this point, the P_pi_ is greater than the P_ni_, so the F_im_ is generated to promote the immune response. The F_im_ curve is divided into two stages, the ascending stage represents the immune process dominated by innate immunity, while the descending stage represents the process dominated by adaptive immunity. After the intersection, the P_ni_ is greater than the P_pi_, so the F_ib_ is generated, resulting in the braking process of immune response. In the process of immune braking, immune memory is formed and tissue repair is carried out. Although the postulate is fictitious, it is very interesting that based on this postulate, the theoretical equations of immunodynamics can be deduced through logical reasoning and mathematical operation, so that the theoretical model of immunodynamics can be established. Based on this theoretical model, *via* factor pair transformation and the introduction of an adjustment coefficient, the equivalent equation that can combine different experimental data is derived. Through a series of postulated equations and the actual operation on the functional data from *Plasmodium* infected mice, a series of hypothetical F_im_ (or F_ib_) curves are obtained. These curves are compared and fitted to the theoretical curve. Then, the fitted curves and their corresponding equations are selected, therefore the practical equations of immunodynamics applicable to type 1 cellular immune response are worked out, namely, equations (7) and (8). Furthermore, the validity of equations (7) and (8) is preliminarily verified by combining functional data. Finally, I attempt to use these practical equations to solve a series of medical problems related to type 1 cellular immune response, such as infection of intracellular pathogens, tumor immunity, and immunotherapy. The practical equations of immunodynamics related to type 2, type 3 cellular, and even humoral immune responses will be left to other investigators.

The four factors of equations (7) and (8), represent the JAK–STAT signal pathway (Gotthardt et al., 2019; Owen et al., 2019; Villarino et al., 2020), and this pathway is now known to play important roles in regulating more than 50 downstream cytokines and growth factors, and is considered the communication center of the immune system, throughout the whole process of the immune response, including innate immunity, adaptive immunity and immune memory. This can be demonstrated by the significant immunophenotypes observed in humans and mice that have lost or gained functional mutations in the genes encoding the JAK–STAT components. The signal transducers and activators of transcription (STATs) in this signaling pathway belong to transcription factors, including STAT1, STAT2, STAT3, STAT4, STAT5a, STAT5b and STAT6, where STAT1 represents a positive immune regulator and STAT3 represents a negative one (Regis et al., 2008; Villarino et al., 2017). STAT2 and STAT4 are similar to STAT1, with the ability to promote Th1 type immune response, while STAT5a, STAT5b, and STAT6 are similar to STAT3, with the ability to promote Th2 type or inhibit Th1 type immune response (Yu et al., 2009). For example, STAT3 expression and activation (phosphorylated form, pSTAT3) are associated with immunosuppression, such as downregulation of IL-12, TNF-α, IFN-β/γ, CD80/86, MHCII, CCL5, CXCL10, up-regulation of IL-6, IL-10, TGF-β, VEGF, PD-1/PD-L1/PD-L2 and CTLA-4. pSTAT3 is also associated with tumor cell proliferation and survival, such as down-regulation of P53 and upregulation of Bcl-XL, Cyclin D1/D2, MYC and Survivin. pSTAT3 is further related to tumor angiogenesis and metastasis, caused by upregulation of MMP2/9, HGF, bFGF, VEGF, HIP-1α, Twist1, Vimentin, and downregulation of AKT, CXCL10, IL-12, IFN-β/γ, and P53. Furthermore, pSTAT3 is related to the number and function of immunosuppressor cells in the tumor microenvironment, such as upregulating the numbers of MDSC, Treg, TAM, and cancer-associated fibroblasts (CAF) and promoting their immunosuppressive function (Yu et al., 2009; Zou et al., 2020). Experimental evidence has shown that pSTAT3 is involved in the formation of immune memory, such as the IL-21-mediated binding of pSTAT3 to CD28, resulting in the transformation of activated CD8^+^ T cells into central memory T cells expressing CD28 and CD62L (Wang et al., 2020). The expression and activation of STAT1 (phosphorylated form, pSTAT1) are involved in a series of actions and functions opposite to those of pSTAT3 (Regis et al., 2008), and therefore pSTAT1 and pSTAT3 constitute an important immunodynamic factor pair. However, this factor pair alone cannot well reflect the characteristics of immunodynamics (as shown in Figure 3 down panel F), and it is necessary to add the factor pair of IFN-γ and TGF-β (as shown in Figure 3 down panel J), namely, equations (7) and (8), to well reflect the characteristics of immunodynamics (as described above). As a pair of immunodynamic factors, the ratio of IFN-γ/TGF-β remained above the baseline level for almost the entire course of innate and adaptive immunity (Figure 3 up and down panel A), while the ratio of TNF-α/IL-10 dropped below the baseline level shortly after innate immunity (Figure 3 up and down panel B). Since the ratio of pSTAT1/pSTAT3 also dropped below the baseline before *Plasmodium* disappeared (Figure 3 down panel F), if the ratio of TNF-α/IL-10 was further introduced into equation (7) to construct a six-factor equation, the validity of the original equation would be damaged (Figure 3 down panel K). Furthermore, IFN-γ is the key cytokine to distinguish type 1 from type 2 and type 3 cellular immune response (Annunziato et al., 2015), and is highly correlated with the signaling pathway of pSTA1 and pSTAT3 (Regis et al., 2008; Yu et al., 2009; Villarino et al., 2017; Zou et al., 2020). Therefore, it is considered that equation (7) and its reciprocal form equation (8), is a good set of practical immunodynamic equations for describing type 1 cell-mediated immune responses.

Based on the above analysis, it may be concluded that the equilibrium and disequilibrium of all type 1 cellular immune responses can be attributed to “normal” and “abnormal” IFNγ/STAT1 and/or TGFβ/STAT3 signaling, such as the acute F_ib_-dominated immune imbalance caused by lethal Py infection, the possible acute Fib-dominated immune imbalance caused by SARS-CoV-2 infection, and the hyperprogression of disease in cancer immunotherapy may all be caused by the acute blockade of the IFNγ/STAT1 signaling, and/or the acute overactivity of TGFβ/STAT3 pathway.

There are already some mathematical models for describing some immune phenomena (Bitsouni and Tsilidis, 2022; Patsatzis, 2022), but they are not the immunodynamic models, and their mathematical reasoning process and expression equations are too complex, it is hard for clinicians to understand, let alone get clinicians to apply them. In stark contrast, the immunodynamic model created here is very simple and can be mastered and applied with just high school mathematics and appropriate knowledge of immunology. However, there may be some concern about whether such a simple model can represent the complex immune phenomena and the mechanisms behind them. It is precisely because the immune system is so complex that a simple model is needed to reveal the nature of immune phenomena. As in the field of physics, the form of energy is also very complex, such as nuclear energy, mechanical energy, chemical energy, internal energy (heat), electrical energy, radiation energy, light energy, biological energy and so on, and between them can also be transformed. However, more than 100 years ago, there was a man who believed that at the deepest level of things there are the simplest principles. This man was Albert Einstein, who developed the theory of relativity through thought experiments and created the most beautiful and simplest equation in the world today, E = mc^2^,^2^ which reveals the nature of energy: Energy is determined only by mass and the speed of light. Similarly, although the immune phenomenon is very complex, there are also only two factors in nature to determine the immune response, namely, P_pi_ and P_ni_ or E_pi_ and E_ni_. “Everything should be made as simple as possible, but no simpler,” “It can scarcely be denied that the supreme goal of all theories is to make the irreducible basic elements as simple and as few as possible without having to surrender the adequate representation of a single datum of experience,” Einstein said in a lecture in 1933.^3^

The theory of immune equilibrium, which attributes all immune factors to the positive and negative immunity and their mutual constraints, has been widely recognized in the field of immunology during the development of more than 100 years. However, it has never been beyond the scope of philosophy, and has always been limited to explain the philosophical significance of complex immune phenomena, so it has never become the core theory of immunology. In essence, the theory of immunodynamics I established is a mathematized immune equilibrium theory, which solves the dilemma that the traditional theory cannot guide individualized medical practice for a long time. The new immunodynamic theory can be used to guide both immunological research and individualized immunotherapy, which may be developed into one of the core theories of immunology in the future. Furthermore, in addition to mathematizing the immune equilibrium theory, the theory of immunodynamics creatively describes the dynamic relationship between innate immunity and acquired immunity by a beautiful kinetic curve, namely, the rapidly rising part (stage) mainly represents the innate immunity while the slowly declining part (stage) mainly represents the acquired immunity. This essential dynamic process has not been described in the traditional theory of immune equilibrium. If the language of immune equilibrium theory is transformed into the language of immunodynamics, then it can be explained that all immune factors are summed up as positive or negative immune power, and after calculation, they are also summed up as immune force or immune braking force. Therefore, all immune responses or immune phenomena are attributable to the contrast or interaction of positive and negative immune power. This is the deepest and simplest basic principle of complex immune phenomena.

In summary, through above-mentioned methodology, the immune equilibrium theory, which has only philosophical significance, is developed into the immunodynamics that can perform actual mathematical operations, can be used to guide personalized medicine.

## Conclusion

In this paper, based on the theory of immune equilibrium, I put forward a series of key immunodynamic concepts and set up their expression formulae through thought experiments, and therefore establish a series of immunodynamic equations to construct the theoretical framework of immunodynamics. Among these equations, all theoretical equations are applicable to all types of immune responses, while the practical equations (7) and (8) are only applicable to type-1 cellular immune response, and have already been preliminarily verified through a minimum of functional data, which are ready to be used in the field of personalized immunotherapy. However, this is only the beginning of immunodynamics research. It is imperative that the two practical equations be applied to the treatment of severe and critically ill COVID-19 to achieve personalized medicine, which could potentially save the lives of a large number of patients and should be acted upon immediately. At the same time, this set of equations should also be applied to cancer immunotherapy as soon as possible to avoid the death event caused by hyperprogression of disease.

## Data availability statement

The datasets presented in this article are not readily available because there were no raw data created in this study. Requests to access the datasets should be directed to chen_xiaoping@gibh.ac.cn.

## Author contributions

The author confirms being the sole contributor of this work and has approved it for publication.

## Conflict of interest

The author declares that the research was conducted in the absence of any commercial or financial relationships that could be construed as a potential conflict of interest.

## Publisher’s note

All claims expressed in this article are solely those of the authors and do not necessarily represent those of their affiliated organizations, or those of the publisher, the editors and the reviewers. Any product that may be evaluated in this article, or claim that may be made by its manufacturer, is not guaranteed or endorsed by the publisher.

## Footnotes

^1^ https://bigthink.com/starts-with-a-bang/meaning-emc2/

^2^ https://bigthink.com/starts-with-a-bang/meaning-emc2/

^3^ https://www.nature.com/articles/d41586-018-05004-4
